# Arginine-to-lysine substitutions influence recombinant horseradish peroxidase stability and immobilisation effectiveness

**DOI:** 10.1186/1472-6750-7-86

**Published:** 2007-12-05

**Authors:** Barry J Ryan, Ciarán Ó'Fágáin

**Affiliations:** 1 School of Biotechnology and National Centre for Sensors Research, Dublin City University, Dublin 9, Ireland

## Abstract

**Background:**

Horseradish Peroxidase (HRP) plays important roles in many biotechnological fields, including diagnostics, biosensors and biocatalysis. Often, it is used in immobilised form. With conventional immobilisation techniques, the enzyme adheres in random orientation: the active site may face the solid phase rather than bulk medium, impeding substrate access and leading to sub-optimal catalytic performance. The ability to immobilise HRP in a directional manner, such that the active site would always face outwards from the insoluble matrix, would maximise the immobilised enzyme's catalytic potential and could increase HRP's range of actual and potential applications.

**Results:**

We have replaced arginine residues on the face of glycan-free recombinant HRP opposite to the active site by lysines. Our strategy differs from previous reports of specific HRP immobilisation via an engineered affinity tag or single reactive residue. These conservative Arg-to-Lys substitutions provide a means of multipoint covalent immobilisation such that the active site will always face away from the immobilisation matrix.

One triple and one pentuple mutant were generated by substitution of solvent-exposed arginines on the "back" of the polypeptide (R118, R159 and R283) and of residues known to influence stability (K232 and K241). Orientated HRP immobilisation was demonstrated using a modified polyethersulfone (PES) membrane; the protein was forced to orientate its active site away from the membrane and towards the bulk solution phase.

Mutant properties and bioinformatic analysis suggested the reversion of K283R to improve stability, thus generating two additional mutants (K118/R159K and R118K/K232N/K241F/R283K). While most mutants were less stable in free solution than wild type rHRP, the quadruple revertant regained some stability over its mutant counterparts. A greater degree of immobilisation on CNBr-activated Sepharose™ was noted with increased lysine content; however, only marginal gains in solvent stability resulted from immobilisation on this latter matrix.

**Conclusion:**

Directional, orientated, immobilisation of rHRP mutants onto an activated, modified polyethersulfone membrane has been achieved with excellent retention of catalytic activity; however, re-engineering of acceptable stability characteristics into the "immobilisation mutants" will determine their applicability in diagnosis and biosensor development.

## Background

**Figure 1:**
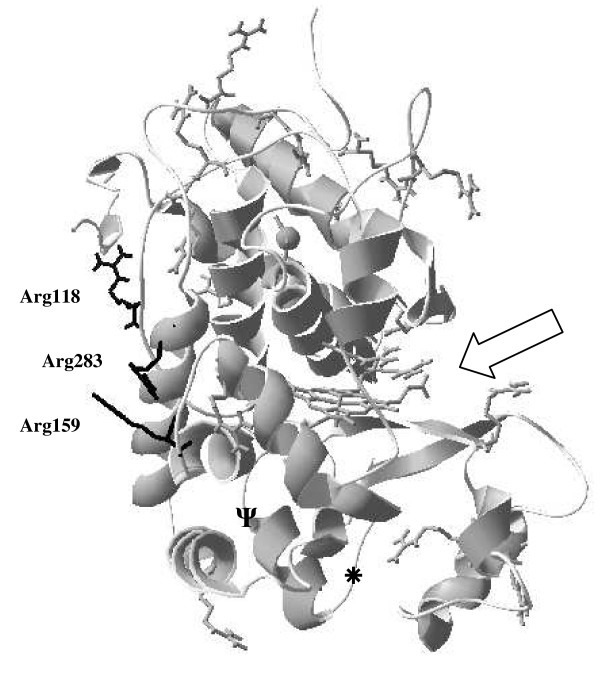
The twenty-one arginine residues in HRP. Those shown in black are the three (R118, R159, R283) selected for mutation to lysine, to enable orientated rHRP immobilisation. The arrow indicates the active site entrance, whilst the substituted lysine residues are also highlighted (232, Ψ; 241, *)

The peroxidase from horseradish roots (*Armoracia rusticana*; HRP, isoform C) is the most widely studied peroxidase, due mainly to its many diverse uses in biotechnology [1]. Several reports of HRP immobilisation exist, commonly employing adsorption as the immobilisation technique [2-4]. Although simple to implement, this approach suffers from several drawbacks, including the removal of adsorbed protein by stringent washing, high contamination (due to non-specific protein binding) and denaturation of proteins adsorbed onto hydrophobic surfaces [5]. Covalent immobilisation of proteins offers a more robust approach. Traditionally, immobilisation of plant HRP relied on periodate oxidation of the enzyme's carbohydrates. In recent years, however, immobilisation technology has developed rapidly, primarily due to the increase in number and range of activated solid supports and the ability to engineer or chemically modify biomolecules to enable easier covalent attachment. Several protein-engineering strategies have introduced reactive amino acids to promote directed protein immobilisation, with lysine being the most common. Abian and co-workers [6] selected asparagine, glutamic acid or arginine residues of Pencillin G Acylase (PGA) for replacement by lysine; these substitutions resulted in improved thermal stability of the immobilised PGA, due to multipoint attachment to a glyoxyl agarose-activated solid phase. HRPC is a highly cationic protein that contains twenty-one arginine residues [7]; however, only Arg residues on the opposite side to the active site entrance were considered for substitution in the present study, as attachment to the solid phase via the "*back*" of the molecule should allow an orientated rHRP immobilisation. This primary condition was supplemented with additional structural criteria (e.g. avoidance of helices, position in relation to the active site entrance, exposure to solvents) and led to the substitution of Arg118, Arg159 and Arg283 (Figure 1) by lysines, thus permitting the investigation of orientated covalent attachment to two activated solid phases, polyethersulfone membrane and CNBr-activated Sepharose™.

## Results

### Immobilisation on PES Membrane

**Figure 2:**
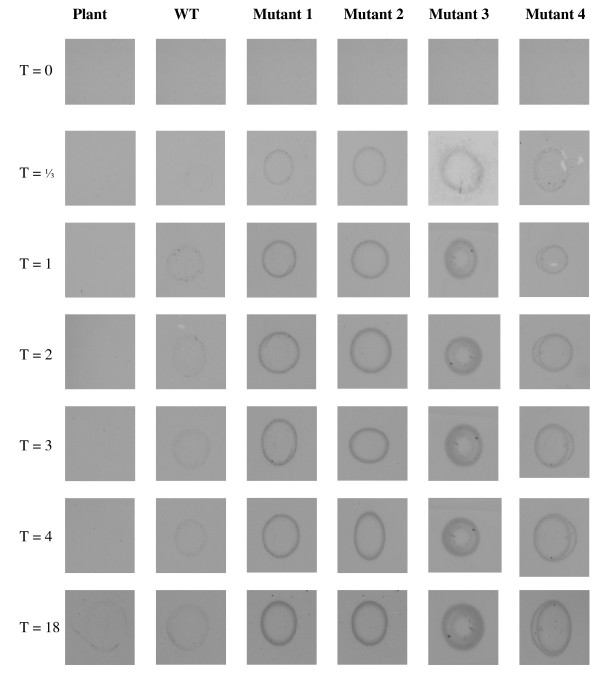
Scanned images of DAB-stained, spotted immobilised rHRP activity. WT, Wildtype; Mutant 1, R118K/R159K/R283K; Mutant 2, R118K/R159K; Mutant 3, R118K/R159K/K232N/K241F/R283K; Mutant 4, R118K/R159K/K232N/K241F. Time points indicated on the left are in hours. DAB coloration indicates peroxidase activity; this was noted within minutes for the immobilisation mutants, whereas plant and wildtype recombinant HRP required a longer time to develop any coloration.

Use of a modified PES membrane permitted successful orientated rHRP immobilisation. Introduction of Arg to Lys substitutions (Mutant 1; Table 1) on the opposite face of the molecule to the active site, together with the removal of wildtype reactive lysine residues (Mutant 3, Table 1; see also Figure 1), forces the protein to orientate its active site away from the membrane and towards the bulk solution phase during covalent immobilisation. Figure 2 demonstrates faster and more intense colour development for Mutant 3 over an eighteen-hour period compared with wildtype. Densitometrical analysis (see Table 2) using Image J software confirms and underscores this point. Equal concentrations of Mutant 1, Mutant 2 (Mutant 1 with reversion at position 238), Mutant 3, Mutant 4 (see Table 1), and wildtype rHRP were applied (see Methods). Increased catalytic activity towards DAB substrate is attributed to optimal orientation of the rHRP molecule (Mutant 3), in contrast to the random orientation of wildtype rHRP or reduced binding capabilities of K283R revertants (Mutants 2 and 4). Furthermore, this experimental set-up also demonstrated reusability. Immobilised Mutant 3 was stained repeatedly (up to three times, data not shown) with TMB (non-precipitating substrate) over a period of several hours, with appreciable catalytic activity noted each time.

**Table 1:** HRP mutants described in this paper

**Name**	**Mutations**
Mutant 1	R118K, R159K, R283K.
Mutant 2	R118K, R159K.
Mutant 3	R118K, R159K, K232F, K241N, R283K.
Mutant 4	R118K, R159K, K232F, K241N.

R, Arg; K, Lys. The numbering system is based on the HRP structure with the PDB accession number 1ATJ.

**Table 2:** % Relative densitometrical analysis of modified PES immobilisation

**Name**	**% Relative Densitometry Values**
Plant	94
WT	100
Mutant 1	127
Mutant 2	140
Mutant 3	163
Mutant 4	138

Plant, WT, Wildtype; Mutant 1, R118K/R159K/R283K; Mutant 2, R118K/R159K; Mutant 3, R118K/R159K/K232N/K241F/R283K; Mutant 4, R118K/R159K/K232N/K241F. % values were calculated using the 'Analyse' function of ImageJ software package [35].

### Stability Properties of Arg → Lys Mutants

Stabilities in free solution of Mutants 1–4 were compared with wildtype. Mutant 1 displayed almost identical thermal and solvent stabilities (except in MeOH) to wildtype rHRP. Mutant 3 was notably less stable (50% decrease in thermal and 20% decrease in MeOH stabilities). This was unexpected, as the chemically modifiable proximal lysine residues (232 and 241, [8]) had been mutated to the stabilising Phe and Asn respectively (Ryan and O'Fágáin, in preparation). Mutant 4 (Mutant 3 with reversion at position 283) displayed a 2.6-fold increase in t_1/2 _at 50°C over Mutant 3 and a 1.3-fold increase versus wildtype. However, Mutant 1 (Arg at position 283) and Mutant 2 (reversion at position 283) displayed very similar thermal stabilities. Solvent stability was also affected by reversion to Arg at position 283: Mutant 2 exhibited a 1/3 increase in C_50 _in DMF, but decreases in DMSO (1/6) and MeOH (1/5) tolerances compared with Mutant 1. Mutant 4 displayed decreased stabilities in DMSO (1/7) and MeOH (1/5).

Interestingly, both reversion mutants displayed greater H_2_O_2 _stability in free solution than their unreverted counterparts (16% for Mutant 2 versus Mutant 1 and 94% for Mutant 4 versus Mutant 3; see Tables 1 and 3).

**Table 3:** Stability Characteristics of HRP Immobilisation Mutants in free solution and immobilised on CNBr-activated Sepharose

	**Thermal Stability**				**Solvent and H_2_O_2 _Stability**			
**Mutant**	**T_50 _**(°C)	***k*-value **(min^-1^)	**SD **(min^-1^)	**t_1/2 _**(min)	**DMSO **C_50_	**MeOH **C_50_	**DMF **C_50_	**H_2_O_2 _**C_50_

**Free Solution**								
WT	50	0.0559	± 0.0034	12	35	53	14	17
Mutant 1	51	0.0535	± 0.0005	12	34	44	12	38
Mutant 2	50	0.0568	± 0.0057	12	30	35	16	44
Mutant 3	48	0.0109	± 0.0007	6	35	45	25	17
Mutant 4	50	0.0445	± 0.0003	17	30	35	16	33
								
**Immobilised**								
WT	-	0.0255	± 0.0023	27	32	40	14	6
Mutant 1	-	0.0223	± 0.0020	3	24	32	17	5
Mutant 2	-	0.0544	± 0.0044	13	28	38	10	5
Mutant 3	-	0.0152	± 0.0017	5	42	43	29	5
Mutant 4	-	0.0491	± 0.0049	14	28	38	15	5

WT, Wildtype; Mutant 1, R118K/R159K/R283K; Mutant 2, R118K/R159K; Mutant 3, R118K/R159K/K232N/K241F/R283K; Mutant 4, R118K/R159K/K232N/K241F. Modelled *k*-values were calculated using the *Enzfitter*™ software package (Version 1.05. Cambridge UK: Biosoft Ltd.; 1987). Values are the mean of three independent determinations in each case. Standard deviations were < 5% for solvent and < 10% for H_2_O_2 _studies. Units of C_50 _are % v/v for solvents and mM for H_2_O_2_.

### Immobilisation on CNBr-activated Sepharose

Increased lysine content correlated with increased immobilisation on CNBr-activated Sepharose: Mutant 4 (3 sites: Lys 118, 159, 174; 42% immobilisation) < wildtype (3 sites: Lys 174, 232, 241; 50% immobilisation) < Mutant 3 (4 sites: Lys 118, 159, 174, 283; 57% immobilisation) < Mutant 2 (5 sites: Lys 118, 159, 174, 232, 241; 69% immobilisation) < Mutant 1 (6 sites: Lys 118, 159, 174, 232, 241, 283; 86% immobilisation). These calculations were based on measured units of activity before and after immobilisation, assuming that no inactivation occurred during the mild immobilisation process. Immobilised wildtype demonstrated more than two-fold thermal stabilisation, whilst immobilised Mutant 2 displayed a marginal 5% increase. Mutant 1 (4-fold), Mutant 3 (1.25-fold) and Mutant 4(1.25-fold) all displayed decreased thermal stability following immobilisation.

Solvent stabilities of wildtype and mutant (both free and immobilised) are set out in Table 2. Small variations (< 2-fold) in solvent stabilities are noted.

All four mutants and wildtype show a dramatically decreased resistance to inactivation by H_2_O_2 _following immobilisation onto CNBr-activated Sepharose, approximating to a basal C_50 _value of 5 mM H_2_O_2 _(Table 3). This contrasts sharply with variations in H_2_O_2 _resistance noted in free solution (Mutants 2, 1 and 4 display a 2.6, 2.2 and 2-fold increase in stability respectively compared to wildtype and Mutant 3; Table 3).

## Discussion

Lopez-Gallego and co-workers [9] recently proposed chemical modification to enrich the protein surface with reactive groups that would facilitate multipoint covalent attachment during protein immobilisation. The present work extends this concept by substituting additional lysines in a specific region of rHRP so as to facilitate directed, multipoint immobilisation onto a commercially available, modified polyethersulfone (PES) membrane. Lysines are often exploited for covalent immobilisation, as they occur frequently in proteins and are active nucleophiles when unprotonated, i.e. at alkaline pH. They are usually located on surface-exposed regions of proteins, due to their charged nature. This characteristic also allows for simple immobilisation reactions, as lysine residues do not require activation prior to immobilisation [10]. To allow directed immobilisation of rHRP to an activated matrix, arginine residues were selected for conservative substitution by lysine. Of the twenty-one arginine residues in rHRP, only three were deemed suitable for the proposed replacements: Arg118, Arg159 and Arg283 (see Methods). These positions are located on the opposite side of the molecule to the active site; hence, immobilisation via these "new" lysines forces the protein to orientate its active site away from the membrane and towards the bulk solution. Maximal colour development was noted during DAB catalysis for Mutant 3 (Lys 118, 159, 174 and 283 available), which can bind only in a uni-directional manner: the reactive wildtype lysines 232 and 241 [8], on a different face of the molecule, have been removed. This directional immobilisation should allow optimal substrate access and product egress.

Although introduction of additional lysines permits directional immobilisation, a stability penalty is incurred. Despite its conservative nature, an Arg-to-Lys substitution can alter hydrogen bonding, as lysine can form only a single hydrogen bond compared with Arg [11]. Reduced hydrogen bonding resulting from the loss of three Arg, coupled with substitution by a bulky aromatic Phe (K232F) residue, may explain the instability of Mutant 3 in free solution. Arg283 is located on the last turn of Helix J [12]. Simple reversion of this residue rescued (or even enhanced) poor thermal and H_2_O_2 _stabilities of the R283K rHRP mutant. Whilst this reversion reduced the number of potential immobilisation sites, the stabilising effect of the K232F and K241N mutations (Ryan and O'Fágáin, in preparation) was evidenced (Table 2; Mutant 4 versus Mutant 2 in free solution). Further protein engineering is required to re-introduce stability to the immobilisation mutants. The work of Strausberg and co-workers [13] has parallels with the present study. They removed a Ca^2+ ^binding loop from subtilisin so as to enhance its stability under metal-chelating conditions. Their initial "loop-less" mutant was much less stable than the wildtype, but it proved possible to "retro-engineer" stability into the mutant, such that it out-performed wildtype under chelating conditions.

Currently, there are very few reports of either direct or random immobilisation of recombinant HRP in the literature. Rojas-Melgarejo and colleagues [2] noted the difficulty in immobilising recombinant HRP expressed in *E. coli *to traditional adsorbent solid phases, since it lacks the carbohydrate residues commonly used for this type of immobilisation. Instead, cinnamic esters were successfully employed (63% immobilisation rate) as an immobilisation platform using physical adsorption in conjunction with glycosylation-independent hydrophobic interaction. Wildtype recombinant HRP immobilised in such a fashion yielded an 8% increase in thermal stability. Abad and co-workers [14] recently described directed recombinant HRP immobilisation via an N-terminal His tag-cobalt (II)-terminated gold nanoparticle interaction. Although no stability data were reported for this method, its potential application in the convenient attachment of rHRP onto gold electrodes for biosensing is obvious. Although our rHRP possesses a C-terminal His Tag, we chose not to exploit this feature for immobilisation and instead adopted the strategy outlined here.

The immobilisation of enzymes can increase protein stability [15]; however, by implementing orientation-dependent protein immobilisation, researchers may not only stabilise a protein, but also promote superior performance in applications such as biosensors. Recent developments in the biosensing area focus on the type of bio-recognition involved in immobilisation. Traditionally, lysines (as in this study) or cysteines can be engineered into a protein backbone to allow for immobilisation; however, research has progressed towards controlled deposition and orientation of immobilised recombinant oxidoreductases for optimal Direct Electron Transfer (DET). The shorter the electron transfer distance, the greater the chance of DET occurring in a biosensor configuration; hence, controlled directional immobilisation of enzymes is important for the progression of third generation biosensors. Typically, physical adsorption is utilised for peroxidase immobilisation onto a metal electrode; however, other methods such as entrapment [16] and direct covalent attachment [17] are employed. Often, these methods lead to the formation of a randomly orientated layer, either on the surface of the electrode (metal) or within cavities (carbon). Physical adsorption may result in enzyme denaturation, due to multiple contacts with the solid phase. Binding of ligands, or substrate/product access and egress, may also be hindered, all resulting in poor bioelectrocatalytic activity [18]. Self-assembled monolayers (SAM) are an attractive alternative for controlled, directed enzyme immobilisation. These highly organised monomolecular layers form spontaneously upon submersion of a solid phase into a solution containing amphifunctional molecules. The lengthy hydrophobic chain of the SAM can be altered and terminated with a functional group that will interact only with a specific residue on an enzyme backbone [19]. In this instance, a site directed mutation could be utilised to dictate the location of the specific residue and, hence, control the orientation of the molecule (e.g additional Cys residue for thiol immobilisation [20]). This concept has recently been adapted for use with chemically-modified plant-derived HRP by Suarez and co-workers [21].

Other methods of immobilisation have developed in recent years, from His-tag chelation to biotin-avidin interaction. Polypeptide scaffolds comprise a topical generic immobilisation method, employing a hydrophobic anchor attached to a leucine-zipper protein capture domain utilised to directionally immobilise several different proteins to a hydrophobic solid phase [22].

Fuentes and colleagues [23] recently noted that the rate of plant HRP immobilisation depended exponentially on the concentration of reactive groups both on the protein and the support. Hence, a protein is mainly immobilised by an area of its surface having the highest density of reactive groups. Lysine is the key amino acid responsible for protein immobilisation onto CNBr-activated Sepharose [24], as reflected in the present study. Only bound Mutant 4 displayed improved stability over free-solution figures, suggesting that multipoint attachment, most evident in the case of Mutant 1, increases strain on the rHRP molecule. This, combined with restricted flexibility, leads to a notable decrease in thermal stability for the mutants. Previous reports of destabilising multipoint immobilisation of glucoamylase also cite increased protein strain as reason for decreased thermal stability [25], while the type of immobilisation resin can also destabilise the immobilised protein [26]. Our results contrast with previous reports of increased thermal stability following multipoint attachment [6]. However, Abian and co-workers [6] focused their mutations on a lysine-rich area of Pencillin G Acylase, which previous chemical modification studies had proven not to be critical for protein stability, and so minimised the chance of a deleterious mutation in this area. This group also utilised a glyoxyl agarose-activated solid phase for protein immobilisation. Plant-derived glycosylated HRP has previously been immobilised onto a variety of solid phases, by a range of different techniques, to increase thermal stability. For example, Rojas-Melgarejo and co-workers [27] noted increased thermal stability (30%) for plant HRP absorbed onto cinnamic carbohydrate esters, similar to that noted in this study for wildtype rHRP (27%), covalently immobilised onto CNBr-activated Sepharose.

The reduced solvent stabilities of the mutants can similarly be ascribed to increased strain and lack of protein flexibility. Due to the confined nature of the immobilised protein, organic solvent molecules that have stripped and replaced water molecules are maintained in close proximity to the protein surface, leading to accelerated protein denaturation [27]. It is well known that many enzymes can function in organic solvents [28]; however, protein denaturation often occurs in water-miscible solvents, by a process referred to as *"water-stripping"*. Gorman and Dordick [29] described how T_2_O (tritiated water) is desorbed from HRP by organic solvents such as MeOH and DMF. DMF, despite its higher dielectric constant, desorbed significantly less T_2_O than did MeOH. The difference is possibly due to structural similarities between MeOH and H_2_O, which allow MeOH to strip and replace water molecules close to the protein surface. There is a major requirement for water for HRP activity [29], so any water removal will have a detrimental effect on enzyme activity. Small losses of solvent stability (e.g. Mutant 3) can be attributed to the reduced number of potential attachments for covalent immobilisation. Hence, the immobilisation of Mutant 3 is not as rigid as that of Mutant 1, permitting some protein flexibility.

Immobilisation on CNBr-activated Sepharose notably decreased H_2_O_2 _tolerance of rHRP wildtype and mutants: the basal C_50 _value was 5 mM H_2_O_2_, much lower than any free-solution value. Previous immobilised HRP H_2_O_2 _tolerance results depend on the type and reactivity of the activated solid phase. Rojas-Melgarejo and co-workers [27] report increased H_2_O_2 _tolerance for HRP adsorbed onto cinnamic carbohydrate esters when the stability analysis was carried out at pH 7.0; at pH 4.5, however, there was a dramatic loss of H_2_O_2 _stability. Those authors believe that reactive oxygen species such as superoxide, generated during H_2_O_2 _catalysis, may have been absorbed by the cinnamic support at pH 7.0 instead of attacking the essential enzyme residues. At lower pH, however, carbonyl groups are oxidised and do not provide a sink for oxidising reactive oxygen species, leading to dramatic enzyme destabilisation [27].

## Conclusion

Directional orientated immobilisation of rHRP mutants onto an activated, modified polyethersulfone (PES) membrane has been achieved with excellent retention of catalytic activity. Despite some loss of thermal, solvent and H_2_O_2 _stabilities both in free solution and following immobilisation onto CNBr-activated Sepharose, orientated rHRP immobilisation has many potential applications including diagnostics and biosensor development. Ultimately, use in these applications will require re-engineering acceptable stability characteristics into the "immobilisation mutants".

## Methods

### Materials

The HRP gene was a generous gift from Prof. Frances H. Arnold (Caltech, CA, USA). The pQE60 vector was purchased from Qiagen (Valencia, CA); XL 10 Gold cells and QuickChange™ Mutagenesis Kit were purchased from Stratagene (La Jolla, CA). Pall *UltraBind*™ PES modified membranes were obtained from AGB Scientific (Dublin, Ireland). All other reagents (including CNBr-activated Sepharose) were purchased from Sigma Aldrich and were of analytical grade or higher.

### Cloning

The HRP gene was directionally cloned into the pQE60 vector as a fusion with the N-terminal pectate lyase (PelB) leader sequence [30] and a C-terminal hexa-histidine purification tag, to generate the plasmid pBR_I. Initially, the PelB leader was cloned via a *Nco *I – *BamH *I double restriction. This introduced a novel *Not *I site 5' to the existing *BamH *I site in the modified pQE60 vector. The HRP gene was then *Not *I – *Bgl *II directionally cloned into a *Not *I – *BamH *I restricted PelB-modified pQE60 vector. This cloning strategy incorporated the poly-His tag, present in the pQE60 vector, at the C-terminus of HRP.

### Bacterial Strains and Plasmids

*E.coli *XL 10 Gold was used as host strain to express the HRP protein. The plasmid (pBR_I), carrying the HRP gene and coding for the HRP fusion protein, was used for expression and site directed mutagenesis.

### Recombinant DNA Techniques

All DNA manipulations were carried out by standard techniques [31]. Site directed mutagenesis was carried out as described by Wang and Malcom [32] utilising the QuickChange™ method. Mutant primers were supplied by MWG-Biotech (Germany). Mutations were confirmed by commercial di-deoxy sequencing (Fusion Antibodies, Belfast, Northern Ireland).

### Expression and Purification

A single cell transformed with pBR_I (or mutant derivative) was grown in LB medium containing 100 μg/mL ampicillin and 2% w/v glucose until the OD_600 nm _reached 0.4; the cells were removed via centrifugation at 2,000 × *g *for 5 min and resuspended in fresh LB supplemented with 100 μg/mL ampicillin, 1 mM δ-ALA and 2 mM CaCl_2_. The cells were then allowed to grow at 30°C, 220 rpm for 16 h. Following overnight expression, the cells were centrifuged at 2,000 × *g *for 5 min and the supernatant was treated with 50% w/v (with respect to the original supernatant volume) ammonium sulphate for 2 h at room temperature. The cells were periplasmically lysed [33] and the periplasmic contents were similarly treated with ammonium sulphate. Proteins precipitated by 50% w/v ammonium sulphate were collected via centrifugation, resuspended in 50 mM phosphate buffer pH 8.0 and dialysed versus the same buffer overnight at 4°C. Sodium chloride (1 M) and GnCl (200 mM) were added to the dialysed fractions, and these latter were subjected to nickel affinity chromatography at room temperature. Sodium acetate (25 mM, pH 4.5) was utilised to elute the bound HRP. The eluted HRP was again dialysed versus 50 mM phosphate buffer pH 7.5 overnight at 4°C, after which the protein was concentrated (Amicon-Plus 20 concentrator tubes), filter sterilised and stored at 4°C.

### Substitution residue selection

Arginine residues in the wildtype were pinpointed for conservative replacement by the reactive side chain lysine, since both have similar size and charge; this should result in minimal secondary structure rearrangement [11]. The twenty-one arginines present in wildtype HRP were viewed in *DeepView *[34] and assessed for suitability based on secondary structure and location. Beta sheet-forming residues were preferred over alpha-helical ones, and surface-exposed residues were selected in preference to buried ones. Only residues on the plane opposite to the active site entrance were considered. These criteria resulted in the selection of R118, R159 and R283 as targets for replacement by lysines (see Figure 1). Of the six wildtype lysines, only 174, 232 and 241 are available for immobilisation under mild conditions [8]. Lys 174, modified to only 20% compared with 100 and 85% for 232 and 241 [8], was left intact throughout this study, but positions 232 and 241 were altered in some of the mutants (see Results section and Figure 1).

#### rHRP immobilisation

##### (a) Pall UltraBind™

Modified PES Membrane possesses activated aldehyde functional groups tailored for amine-based covalent immobilisation. HRP (plant or recombinant) was resuspended in 50 mM sodium phosphate buffer, pH 7.5 (enzyme concentration 30 pM), and directly spotted onto the activated membrane; the latter was allowed to air-dry completely at room temperature for 10 min. The remaining binding sites were then blocked with 1% w/v solution of food-grade non-fat dry milk in 50 mM sodium phosphate buffer, pH 7.5, for 1 hour at room temperature. The membrane was then washed with 50 mM sodium phosphate buffer, pH 7.5, and allowed to air-dry completely at room temperature for 10 min. DAB (precipitating chromogen) and TMB (non-precipitating chromogen) were utilised to locate immobilised HRP prior to imaging.

##### PES Immobilised HRP-Imaging

Developed membranes were imaged with a Hewlett Packard Scanjet 5590, connected to a Dell Optiplex Computer with the following parameters: output type, true colour (24 bit); output scale, 100%; output resolution, 2400 dpi; sharpen level, extreme; scan form, scanner glass; highlights, -100; shadows, -100; and midtones, -100. True colour images were converted to greyscale using Hewlett Packard Scanjet 5590 image software.

#### Image J Analysis

Saved greyscale images were opened in Image J software [35]. The background was subtracted from the image using the Process>subtract background function. A rolling ball radius of 50 and a white background were selected. The image threshold was also automatically adjusted to black and white. The image was cropped and desitometrical analysis carried using the 'Analyse' function. The results were expressed as a %age of the wildtype value.

##### (b) CNBr activated Sepharose^® ^4B

HRP was suspended in coupling buffer (0.1 M NaHCO_3_/Na_2_CO_3 _containing 0.5 M NaCl, pH 8.5; enzyme concentration 30 pM). CNBr-activated Sepharose^® ^4B was aliquoted into a clean purification column (2 × 8 cm), then washed and swollen in cold 1 mM HCl for at least 30 min. The resin was then washed with 10 column volumes of distilled water and, finally, with coupling buffer. Immediately, the resin was transferred to the solution containing HRP and mixed on an end-over-end rotator for 2 hours at room temperature. After coupling, any unbound protein was removed using several washes of coupling buffer. Any remaining unreacted groups on the Sepharose particles were blocked with 0.2 M glycine, pH 8, for 2 hours at room temperature. Extensive washing with high (A: 0.1 M NaHCO_3_/Na_2_CO_3 _buffer containing 0.5 M NaCl, pH 8.5) and low (B: 0.1 M acetic acid-sodium acetate buffer, pH 4) pH buffers, A and B respectively, removed the glycine and consolidated the HRP-to-resin covalent bonds.

##### H_2_O_2 _and Thermal Tolerance Analysis

H_2_O_2 _stability of recombinant HRP, and mutant variants, was determined as described in ref. [36]. In brief, rHRP (360 nM in 50 mM phosphate buffer, pH 7.0) was incubated with increasing concentrations of H_2_O_2 _(0–100 mM). H_2_O_2 _concentrations were determined spectrophotometrically at 240 nm using 43.6 M^-1^cm^-1 ^as the extinction coefficient [37]. Samples were exposed to the relevant H_2_O_2 _concentration for 30 min at 25°C in a temperature-controlled waterbath. Residual catalytic activities were then measured (below) (note that the H_2_O_2 _concentration used with a reducing co-substrate in the activity assay below was significantly lower than the concentration of H_2_O_2 _as sole substrate that led to 50% inactivation (C_50_) [38]). The thermal stability parameters of recombinant HRP and mutant variants were determined as follows. A single peroxidase stock solution was placed for 10 min at progressively increasing temperatures, at which time aliquots were withdrawn, chilled on ice and eventually assayed under optimal conditions. This procedure gives T_50_, or the temperature of 50% inactivation. For thermal inactivation at a constant 50°C, a single peroxidase stock solution (room temperature) was plunged into a waterbath held at 50°C and aliquots were withdrawn every minute for ten minutes onto ice and later assayed under optimal conditions. The solvent parameters of recombinant HRP and mutant variants were determined as described for plant HRP [39] except that the solvents employed were methanol, dimethylsulfoxide and dimethylformamide.

Following temperature, solvent or H_2_O_2 _incubation, aliquots (50 μL) were withdrawn and the remaining catalytic activities were assayed using a standard microtitre-based TMB assay. This comprised 150 μL of 32 mM TMB substrate (in 100 mM citric acid buffer, pH 5.5, containing 3 mM H_2_O_2_) and 50 μL of rHRP in each well. The microplate was shaken as the initiating enzyme was added and the absorbance at 620 nm was recorded after 6.5 min reaction time. The C_50 _value (mM H_2_O_2 _or % v/v solvent where 50% of maximal HRP activity still remains) was utilised to compare H_2_O_2_/solvent stabilities across the mutant matrix, whilst the *t*_1/2 _(half-life) was employed to compare mutant thermal stabilities (see Table 3).

## Abbreviations

ABTS, 2,2'-azino-bis(3-ethylbenzthiazoline-6-sulfonic acid; δ-ALA, delta aminolevulinic acid; C_50_, concentration of H_2_O_2 _leading to 50% inactivation after 30 min at 25°C; DAB, diaminobenzidine; DMF, dimethylformamide; DMSO, dimethylsulfoxide; dpi, dots per inch; HRP, horseradish peroxidase isoenzyme C; GnCl, guanidine hydrochloride; LB, Luria-Bertani medium; MeOH, methanol; rHRP, recombinant horseradish peroxidase isoenzyme C; PES, polyethersulfone; t_1/2*app*_, apparent half-life; T_50_, temperature leading to 50% inactivation after 10 min; *t*_1/2_, half-life; TMB, 3,3',5'5-tetramethyl benzidine; v/v, volume per volume; w/v, weight per volume.

## Authors' contributions

BJR designed this study and participated in its conception. BJR also carried out all experimental work, data collection, analysis and worked jointly on manuscript drafting.

CF conceived the study, participated in its design and co-ordination, and worked jointly on manuscript drafting.

Both authors read and approved the final manuscript.

## References

[B1] Ryan BJ, Carolan N, O'Fágáin C (2006). Horseradish and Soybean Peroxidases: Comparable Tools for Alternative Niches?. Trends in Biotechnology.

[B2] Rojas-Melgarejo F, Marín-Iniesta F, Rodríguez-López JN, García-Cánovas F, García-Ruiz PA (2006). Cinnamic carbohydrate esters show great versatility as supports for the immobilization of different enzymes. Enzyme and Microbial Technology.

[B3] Fuentes M, Mateo C, Guisan JM, Fernandez-Lafuente R (2005). Preparation of inert magnetic nano-particles for the directed immobilization of antibodies. Biosensors and Bioelectronics.

[B4] Ferapontova E, Dominguez E (2002). Adsorption of differently charged forms of horseradish peroxidase on metal electrodes of different nature: effect of surface charges. Bioelectrochemistry.

[B5] Cretich M, Damin F, Pirri G, Chiari M (2006). Protein and Peptide arrays, Recent trends and new directions. Biomolecular Engineerin.

[B6] Abian O, Grazu V, Hermoso J, Gonzalez R, Garcia JL, Fernandez-Lafuente R, Guisan JM (2004). Stabilization of penicillin G acylase from *Escherichia coli*: site-directed mutagenesis of the protein surface to increase multipoint covalent attachment. Applied Environmental Microbiology.

[B7] Welinder K (1979). Amino Acid sequence studies of HRPc. European Journal Biochemistry.

[B8] O'Brien AM, O'Fágáin C, Nielsen PF, Welinder KG (2001). Location of Crosslinks in chemically stabilised HRP. Implications for design of crosslinks. Biotechnology and Bioengineering.

[B9] Lopez-Gallego F, Montes T, Fuentes M, Alonso N, Grazu V, Betancor L, Guisan JM, Fernandez-Lafuente R (2005). Improved stabilization of chemically aminated enzymes via multipoint covalent attachment on glyoxyl supports. Journal of Biotechnology.

[B10] O'Fágáin C, O'Fágáin C (1997). Protein stability and its measurement. Stabilising protein function.

[B11] Betts MJ, Russell RB, Barnes MR, Gray IC (2003). Amino acid properties and consequences of substitutions. Bioinformatics for Geneticists.

[B12] Gajhede M, Schuller D, Henriksen A, Smith A, Poulos TL (1997). Crystal structure of HRPC at 2.15 Å resolution. Nature Structural Biology.

[B13] Strausberg SL, Alexander PA, Gallagher DT, Gilliland GL, Barnett BL, Bryan PN (1995). Directed evolution of a subtilisin with calcium-independent stability. Biotechnology.

[B14] Abad JM, Mertens SFL, Pita M, Fernandez VM, Schiffrin DJ (2005). Functionalization of Thioctic Acid-capped gold nanoparticles for specific immobilisation of Histidine-tagged proteins. Journal of the American Chemical Society.

[B15] Mateo C, Abian O, Fernandez-Lafuente R, Guisan JM (2000). Increase in conformational stability of enzymes immobilised on epoxy-activated supports by favouring additional multipoint covalent attachment. Enzyme and Microbial Technology.

[B16] Yang M, Yang Y, Shen G, Yu R (2004). Bienzymatic amperiometric biosensor for choline based on mediator thionine in situ electropolymerized within a carbon paste electrode. Analytical Biochemistry.

[B17] Castillo TJ, Sotomayor MdPT, Kubota LT (2005). Amperiometric biosensor based on horseradish peroxidase for biogenic amine determinations in biological samples. Journal of Pharmaceutical and Biomedical Analysis.

[B18] Freire RS, Pessoa CA, Mello LD, Kubota LT (2003). Direct Electron Transfer: An approach for Electrochemical Biosensors with higher selectivity and sensitivity. Journal of the Brazilian Chemical Society.

[B19] Gilardi G, Fantuzzi A (2001). Manipulating Redox systems: Applications to nanotechnology. Trends in Biotechnology.

[B20] Ferapontova EE, Schmengler K, Borchers T, Ruzgas T, Gorton L (2002). Effect of Cysteine mutations on direct electron transfer of HRP on gold. Biosensors and Bioelectronics.

[B21] Suarez G, Jackson RJ, Spoors JA, McNeil CJ (2007). chemical introduction of disulfide groups on glycoproteins: a direct protein anchoring scenario. Analytical Chemistry.

[B22] Zhang K, Diehl MR, Tirrell DA (2005). Artificial Polypeptide Scaffold for Protein Immobilization. Journal of the American Chemical Society.

[B23] Fuentes M, Mateo C, Fernández-Lafuente R, Guisán JM (2006). Detection of Polyclonal Antibody Against Any Area of the Protein-Antigen Using Immobilized Protein-Antigens: The Critical Role of the Immobilization Protocol. Biomacromolecules.

[B24] Wilcheck M, Miron T (2002). Orientated versus random protein immobilisation. Journal of Biochemistry and Biophysical Methods.

[B25] Gerasimas VB, Chernoglazov VM, Klesov AA (1980). Effect of progressive chemical modification on the activity and thermal stability of soluble and immobilized glucoamylase. Biokhimiia.

[B26] Nys PS, Savitskaia EM, Shellenberg NN (1977). Penicillin amidase from *E. coli*. A comparative study of the stability of penicillin amidase immobilized by various means. Antibiotiki.

[B27] Rojas-Melgarejo F, Rodriguez-Lopez J, García-Cánovas F, García-Ruiz PA (2004). Immobilisation of Horseradish peroxidase on cinnamic carbohydrate esters. Process Biochemistry.

[B28] Ogino H, Ishikawa H (2001). Enzymes that are stable in the presence of organic solvents. Journal of Bioscience and Bioengineering.

[B29] Gorman LAS, Dordick JS (1992). Organic-solvents strip water off enzymes. Biotechnology and Bioengineering.

[B30] Lei SP, Lin HC, Wang SS, Callaway J, Wilcox G (1987). Characterisation of the *Erwinia carotovora *pelB gene and its product pectate lyase. Journal of Bacteriology.

[B31] Sambrook J, Fritsch EF, Maniatis T (1989). Molecular Cloning-A laboratory Manual.

[B32] Wang W, Malcom BA (1999). Two-stage PCR protocol allowing introduction of multiple mutations, deletions and insertions using QuickChange™ site-directeed mutagenesis. BioTechniques.

[B33] French C, Keshavarz-Moore E, Ward JM (1996). Development of a simple method for the recovery of recombinant proteins from the *E.coli *periplasm. Enzyme and Microbial Technology.

[B34] Guex N, Peitsch MC (1997). SWISS-MODEL and the Swiss-PDB Viewer: An environment for comparative protein modelling. Electrophoresis.

[B35] Abramoff MD, Magelhaes PJ, Ram SJ (2004). Image Processing with Image J. Biophotonics International.

[B36] Arnold FH, Lin Z (7127). Expression of Functional Eukaryotic Proteins. Worldwide Patent: PCT/US99/1 WO 00/006718, 2000. California Institute of Technology.

[B37] Hernández-Ruiz J, Arnao MB, Hiner ANP, Garcia-Cánovas F, Acosta M (2001). Catalase-like activity of horseradish peroxidase: relationship to enzyme inactivation by H_2_O_2_. Biochemical Journal.

[B38] Ryan BJ, Ó'Fágáin C (2007). Effects of single mutations on the stability of Horseradish Peroxidase to hydrogen peroxide. Biochimie.

[B39] Miland E, Smyth MR, Ó'Fágáin C (1996). Increased thermal and solvent tolerance of acetylated horseradish peroxidase. Enzyme and Microbial Technology.

